# Spiritual Journey in Mothers’ Lived Experiences of Caring for Children With Autism Spectrum Disorders

**DOI:** 10.5539/gjhs.v7n6p79

**Published:** 2015-03-30

**Authors:** Abbas Heydari, Laleh Hosseini Shahidi, Ali Mohammadpour

**Affiliations:** 1 Evidence-Based Caring Research Center, Department of Medical-Surgical Nursing, School of Nursing and Midwifery, Mashhad University of Medical Sciences, Mashhad, Iran; 2 School of Nursing and Midwifery, Mashhad University of Medical Sciences, Mashhad, Iran; 3 Gonabad University of Medical Sciences, Gonabad, Iran

**Keywords:** Autism Spectrum Disorders, mother’s experiences, caring, hermeneutic, spiritual journey

## Abstract

**Introduction:**

Helping mothers who have children with autism spectrum disorders requires understanding of their lived experiences. This study aims to uncover the spiritual journey as a main theme in Iranian mothers’ experiences.

**Method:**

This hermeneutic phenomenological study is a part of a larger study undertaken for partial fulfillment of the requirement for PhD dissertation in nursing. The main study was performed on 18 cases of Iranian mothers, with experience of caring for a child with an autism spectrum during 2011-2012. They were selected based on purposeful sampling method. Semi -structured interviews for data collection were used. Data analyses were done with the interpretative method.

**Results:**

*Spiritual journey* is one of the main themes of the phenomenon under study in the original project. It consists of three sub -themes each of which supported by a number of common meaning. The sub-themes and their common meanings in parenthesis are (1) *Descent: wondering between what is and what will be* (having sorrowful tale, unanswered question, escaping from reality, losing hope) (2) *Connecting to deity: reflection on the failure in her struggle* (gratefulness, surrendering to god, having the divine test) (3) *Ascent: helping her child is becoming all of the mother’s life* (to rescue, being hopeful, listening to her inner voice).

**Conclusion:**

This research concluded that caring for the autistic children led mothers’ lives to raise spirituality and enabled them to help their children and themselves, to grow and be refined in this process.

## 1. Introduction

Autism spectrum disorders (ASD) are one of the chronic developmental disorders that are spread in all of the world’s population (Orme, 2005). ASD refer to a group of developmental disorders with similar symptoms such as Autistic syndrome, Asperger’s syndrome, Pervasive Developmental, and Childhood Disintegrative Disorders (American Psychiatric Association, 2013). Today, we face the increasing prevalence of autism spectrum up to 6.0% in the world that can be future challenges for public health organization (Smith, 2010). ASD are apparent in early childhood and present a wide variety of crippling, lifelong disabilities in social interactions, verbal and nonverbal communications, and other behavioral domains (Halfon, Houtrow, Larson & Newacheck, 2012).

For the parents the ideal image of having a normal child after discovering a developmental disorder is ruined (Orme, 2005). Children with autism spectrum disorder require more care than children with other chronic developmental disorders because of their special conditions (Sawyer et al., 2010). After diagnosis, parents have to adapt with many changes that alter their lives priorities (Lin, Tsai, & Chang, 2008). In comparison with other family members mothers are particularly affected by these challenges and adjustments, both at home and in the community (Halfon, Houtrow, Larson & Newacheck, 2012). Being a mother of a child with ASD is not easy because these children have very distinct needs and require more care, guidance, supervision, and support (Smith, 2010). Obviously, caring for autistic children because of their particular circumstances is a strain situation for mothers (Hoogsteen & Woodgate, 2013). Indeed, mothers as primary care givers endure more problems for caring of autistic spectrum children (Eisenhower & Blacher, 2006; Sari, Base & Turan, 2006).

The mothers role to care and cure in children with ASD is known and this fact has revealed the necessity of supporting mothers in taking care and adapting with this condition (Marshall & Long, 2010). It is obvious mothers of children with autism spectrum need more professional support and now- a days we can see more supporting programs for them in the world (Samadi & McConkey, 2011).

Mothers’ lives can alter when they faces a serious accident or an incurable disease like autism and taking care of children with autism spectrum can be directly affected by mothers’ life context (Shaked & Bilugrapplin, 2006). ASD put mothers in difficult circumstances so the mothers’ lives are influenced and that can affect aspects of mothers’ holism (Peres, Moreira- Almeida, Nasello & Koenig, 2007). Mothers try to cope with this difficult and painful event with seeking support from different resources. Also, facing this event gives an opportunity to think more the meaning of life for mothers (Culliford, 2002). Recently, many studies have tried to find a way to reduce effects of children with ASD on family members especially mothers (Ogston, Mackintosh, & Myers, 2011; Bilgin & Kucuk, 2010).

Mothers’ stories have led professionals to realize mothers’ abilities to accept their children’s disorder and form strong mother-child attachments necessary for normal healthy development. Indeed, nursing implementation and helping mothers depends on the understanding of mother’s lived experiences in caring autistic children (Orme, 2005). This only will be possible by keeping close contact with mothers and exploring their feelings, attitudes, and perspectives, and their children’s disorder with qualitative research. The authors believe that understanding the meanings of the mothers’ experiences in taking care of autistic spectrum children can provide a good guidance for health care services that provide necessary care and identify basic needs based on mothers’ perceptions.

This phenomenon, caring for autistic child, is common in the Iranian culture. Iranian’s mothers are traditional primary caregivers and more likely to be in charge of the child care than others for an autistic child. Although the challenges of caring for ASD children have mostly been considered in Iran but there aren’t enough data about the lived experiences of mothers who are involved with caring for these children. Indeed, mother’s experiences usually are neglected by Iran’s health care system (Samadi & McConkey, 2011). Since the existing knowledge about this phenomenon in Iran is still ambiguous, it needs to be studied more.

This study has been done in order to understand Iranian mother’s lived experiences in caring for a child with autism. Also, researchers hope the findings of this study will provide valuable information for Iranian mothers and professionals who interact with mothers in promoting more effective care for children with ASD.

## 2. Material and Method

### 2.1 Study Design

This research is a phenomenological hermeneutic study. It is also part of a larger study for a doctoral dissertation in nursing with the purpose of understanding the lived experiences of Iranian mothers caring for children with ASD. The spiritual journey is one of the major themes of mothers caring experiences. Phenomenological approach is suitable for this study because it pursues the human experiences within the context of people daily lives. Human responses to health and illnesses are different, due to uniqueness of each person. Based on humanistic theory in nursing, appropriate nursing intervention could be achieved through interpretative study results. Actually, most situation need to understood and interpreted by nurses more than they need nursing intervention (Orme, 2005). Nurses need the knowledge of “human being” in order to use it for developing knowledge and better caring of clients (Dowling, 2007). The stories of mothers should be investigated for realizing the inherent experiences that they experience in the struggle with their child’s disorder.

### 2.2 Participants’ Profile

Participants were selected by purposeful sampling with maximum variation. The sampling went on until data saturation accrued up to the time when there was no new idea available. They were chosen from Community of Supporting Autistic Children, public and private centers affiliated with the education and training organization of exceptional children in Mashhad. The inclusion criteria were mothers’ who each had one ASD child that at least caring for them one year and were inclined to take part in this study. Mothers with drug abuse, divorced, history of psychiatric disorders, and mothers who had another child with different diseases were omitted. Participants of this study contained 18 mothers between 25 to 48 years old and all of them were Muslim, spoke Persian, married and lived with their husbands in Mashhad city. Also their patient children were between 4 to 11 years and their disease had been diagnosed in the age range between 5 years and three month to 6 years. Most of the mothers mentioned that their child’s disease was autism and most of these children were male. Participants’ profile in this study has been shown in Table 1.

**Table 1:** Participants’ profile

participants	Age	Education	Occupation	Economic status	Sex of children	Child’s disorder	Number of children
1	48	Bachelor’s	Full time	Good	M	Autism	3
2	44	Senior high	Housewife	Med	M	Autism	2
3	24	Diploma	Housewife	Poor	M	Autism	1
4	43	Literate	Housewife	Poor	M	Asperger	2
5	25	Literate	Housewife	Poor	F	Autism	1
6	24	Bachelor’s	Housewife	Good	M	Asperger	1
7	31	Literate	Full Time	Good	M	Autism	2
8	45	Literate	Part time	Med	M	Autism	1
9	26	Junior high	Housewife	Poor	F	Autism	1
10	28	junior high	Part time	Poor	M	Autism	3
11	32	Diploma	Housewife	Poor	M	Pervasive Developmental	2
12	30	Bachelor’s	Part time	Med	F	Autism	1
13	48	Bachelor’s	Full time	Good	M	Autism	3
14	44	Senior high	Housewife	Med	M	Autism	2
15	26	Diploma	Housewife	Poor	F	Autism	3
16	43	Diploma	Housewife	Poor	M	Asperger	2
17	31	Diploma	Housewife	Poor	M	Autism	2
18	35	Bachelor’s	Housewife	Good	M	Autism	1

### 2.3 Data Collection

Deep, semi-structured, and face to face interviews were used to collect data in this study. Data collection took place from September to December 2012.The interviews were done with following the interview guide. Interviews were started with an open ended-question such as, would you please tell me about your life story for caring an autistic child? What is the meaning of taking care of an autistic child for you? Does facing this difficult and painful event give an opportunity to think more and give more meaning to your life? All interviews had taken by one researcher who was a nursing PhD student. Interviewer used probing technique in order to clarify some parts of dialogue and to take deeper data during interviews: What is the meaning of what you said. Also field notes were made about emotions encountered during the interviews, and any nonverbal behaviors of the participants. Each participant has been invited to interview two or three times in different situations. Interviews carried out at the location where participants would prefer. These interviews lasted between 40 and 70 minutes. Recorded interviews were transcript verbatim as soon as possible.

### 2.4 Rigor and Trustworthiness

Rigor and trustworthiness of qualitative data in this study was guaranteed by Lincoln and Gubas’ evaluation criteria (Paton, 2002). Credibility was handed by participant verification and peer debriefing (member check). Dependability was considered through constant analysis and prolonged engagement with participants (more than one year). Also, peer consultation was performed with the purpose of attaining deep data and common understandings. The researcher’ effect was prevented by placing participants in comfortable situations without any compulsion. Confirm- ability of the findings was considered through the control of an external examiner and also, the process of coding and categorizing were confirmed by research group. To reach transform-ability all the data in this study was collected precisely so that another person reviewing this research could easily do a follow up.

### 2.5 Data Analysis

Data were analyzed based on interpretative method of Diekelmann, Allen, and Tanner (1989) that has been applied frequently by nursing researchers (Dikelman, Allen, & Tanner, 1989). Regarding this method, data collection and analysis were simultaneously carried out and hermeneutics’ circle was considered. To categorize data researcher used the MAXQDA 2007. In during the data collection and analysis, selected first interview was analyzed to identify missed or obscure parts, potential themes, and any disagreements. Next, the discussions were carried out about the need for much clarification. Later interviews were conducted by highlighted points from the first interview. After each interview, new questions came up and they were added to the fowling the interview’s guide or modified it slightly. In the first step, all manuscripts derived from interviews in order to attain a general notion were read and reread by the researchers and interpretational abstracts were written for each text. During the next step, the research group found out the themes, which were disclosed in the participants’ dialog. Also, previous mothers’ experiences to interpret were considered. Written interpretation was developed along with interviews and rewriting the manuscripts. Then, the whole paragraph would be reviewed and a general theme would be derived from the texts. These themes were discussed and confirm by the research group. Also, any conflict in opinion among researchers was considered. At this stage the process of assigning code names was fulfilled. Researchers compared the themes to find similarities and differences between derived meanings from all texts by revising the main texts. The achieved text was verified by the researcher group. Interpretive writings were discussed continuously and interpretive reports of the group were refined.

### 2.6 Ethical Considerations

This study was approved by the ethics committee of Mashhad University of Medical Sciences (Ref. 900550). Written consent was obtained from all participants especially for voice record. Participants were also assured that their information was confidential and they would have the right to withdraw from the study at any time. Audio files were saved with nicknames and were transformed to text by the researchers and only ones that had access to the computer files were the researchers.

## 3. Results

The major theme of this spiritual journey was emerged from processing of data analysis during the fusion of horizons between researcher and participants and it helped to understand the meaning of mother’s experiences in this study. It was explained in terms of three sub-themes each of which was supported by common meanings. *Descent*: wondering between what is and what will be (having sorrowful tale, unanswered question, escaping from reality, losing hope) (2) *Connecting to deity*: reflection on the failure in her struggle (gratefulness, surrendering to god, having the divine test) (3) *Ascent*: helping her child is becoming all of the mother’s life (to rescue, being hopeful, listening to her inner voice). The summery of sub-themes and common meanings have been shown in Table 2.

**Table 2:** Summery of themes and of sub- themes

Major theme	Sub-themes	Common meanings
	1) Descent: wondering between what is and what will be	- Having sorrowful tale - Unanswered question - Escaping from reality - Losing hope
Spiritual journey	2) Connecting to deity: reflection on the failure in her struggle	- Gratefulness - Surrendering to God - Having the divine test
	3) Ascent: helping her child is becoming all of the mother’s life	- To rescue - Being hopeful -Listening to her inner voice

### 3.1 Descent: Wondering Between What Is and What Will Be

The journey of spirituality that the mothers in this study were began with the wondering between what is and what will be. Pain and suffering related to the life and care of autistic children, have broken down the world of mothers in this study and it changed the meaning of their lives. Endless grief and sorrow of all the participants began just after diagnosis of disability in children and it was developed in all aspects of their lives. Although most mothers were aware of the disorder of their children but they could not believe it in their hearts and this would cause them to escape from the reality of the child’s disorder. Also, most mothers were faced with an obsessing unanswered question “why me?” They followed the cause of this unfortunate disorder and an explanation for this situation, but the more they searched the less they could find and no logic could explain this question. Also, they lost their hope and seemed very confused because of not being aware of what will happen to their children.

Some of the participants explained their frustration as follows: “when I knew my child was ill and not good, you don’t know how much I was sad and I got mad. I always asked myself and God why my kid should be like this. Why my baby should get through this way. It was an unanswered question. I couldn’t accept it. I realized that my child has a problem, but I did not admit. How should I accept? So it’s hard to bear. So, my child had a problem but I couldn’t help him to get better and continue to live like this until the end. To be honest it couldn’t be accepted by my soul.”(p5)

“I cried so much when I realized my son has autism. Due to this subject I was so upset. What am I doing that my child likes this? I am suffering from this issue. I understood that my baby getting sick and have trouble but I didn’t want to accept. I tried not to say my child was ill. I did not want to admit it but I had. I had not any other choices. Well, I though it is better that I don’t say anything about it. Maybe his disease gets better someday so what should I say?” (p1)

“I had become so tired of the things I would do for him. Everything done for a sick child is useless. We do everything without any result and hope. I do not have any cheer. Totally I reached the point where I do not like to train him. I do not know anything about the future; my child will not be healthy. I have not any hope for the future.”(p3)

### 3.2 Connecting to Deity: Reflection on the Failure in Her Struggle

Mothers, in this study through the initial involvement, continued their spiritual journey with connecting to deity. They didn’t know how to manage their challenges and after reflecting on their failure for caring of their children they moved toward choosing spirituality and their religious practice to help them. Indeed, they came along with this disorder and becoming calm by entrusting their hearts to God. Most of mothers considered their child’s disorder as a God willing and because of different reasons such as having a normal appearance, relative abilities of an autistic child in comparison with other developmental disorders and also in line with the religious’ beliefs of being grateful, they thanked God even in difficult situations. Some of the participants considered the existence of child as a divine test in their lives. They also considered it as a duty that was given to them by God. They believed that their will is too weak to be able to intervene in the God’s will, and resorted to God in most difficult situations.

As it was revealed in participant’s sayings “Most of times I talk with God. Maybe God wanted him to be like this. The only thing that helps me is relaying on God right now. Faith in God helped me to get along with the problems. We should do our best and leave the rest to God.” (p2)

“I always confided with God, how much that I pray. When I am sad, I pray, I send blessings. I am trying to help myself in this way and get along with my sadness. I told myself that it is not a big problem. As God gave this disease to my child will give her the cure too. Maybe God want to examine me. I always thank God because of child’s normal appearance and walking and doing routines.”(p4)

“My faith always has helped me to live during hard times; I am satisfied with God’s will. My beliefs give me the power to face with everything that occurs every day. We should be thankful for everything even when everything is bad.” (p7)

### 3.3 Ascent: Helping Her Child is Becoming All of the Mother’s Life

The participants continued their path arising to spirituality level. That means they have considered the process of caring the child as a responsibility that was given to them in their life and none of them replace caring the child with surrender to God. Although most of them knew that their children’s disorder will be incurable, they did not stop their efforts and consider the only forwarded way as helping their children. Interestingly, the feeling of doing so much effort for nothing and disappointment revealed in only of a mother’s comments. However most of them believed that they should do something to help the children to reach the destination peak in any possible way. Also, facing difficulties could not take mother’s hope for better future. Also, mothers of this study that were offered by a little professional support and assistance for caring the sick child, took care of their children according to their internal feelings, in other words inner voice would be the guide for them in terms of caring for the child.

“I told myself that I should do something beneficial, I make a strong decision about doing something and stop wailing. It couldn’t be possible not to care about it.” (p6). “The only thing I can do now is to help him. I think what should I do for his problem? What can I do for him to get better?”(p9)

“With all these difficulties the future will be bright. I imagine good future for my son. He is going to school and we are living together and I hope for the best.” (p5)

“Although I still have hard days and I should fight, I hope we will pass these days. My faith helped me to be strong and tolerate. God wants him to be sick.” (p7)

“I feel something in my thoughts. My sense told me: you should train her in this way. I feel I should train her as much as possible. It seems that, I know how to train her to reach better results. I understood lots of things about her trainings myself.” (p1)

“I understand and feel some sort of things. Nobody told me what I should do. It was only my sense. I often understand how to like the time that you have special feeling in your mind. I feel it will be better if I do it in this way so I did and reached good results after that.” (p2)

## 4. Discussion

Traumatic events of life, like the unexpected birth of a child with special needs not only forces mothers to face with thinking about the meaning of life (Frankl, 2011), but also it provided an opportunity to attaining more spirituality (Samios, Pakenham, & Sofronoff, 2008). The same as findings of some studies for mothers in this study caring for children with ASD was a painful and a troublesome experience (Lecavalier, Leone, & Wiltz, 2006; Firat, Diler, Avci & Seydaoglu, 2002). Our participants, after diagnosis of their children’s disorder, started to search for explanation for this problem but they didn’t find anything that matched with fewstudies (Oprea & Stan, 2012; Lutz, Patterson & Klein, 2012).

Indeed, the pain and suffering along with life and taking care of a child with ASD that accompanied by disassemble the mothers’ world in this study and they were put against a different meaning of life. Mothers in the process of raising and caring for their children reached deeper meanings of their lives. Also, they were able to continue their lives only by taking a path of spirituality to move forward even though facing many obstacles on their way to continue their lives.

Although only small group of mothers pointed out positive effects of taking care of ASD children (Oprea & Stan, 2012), most participants in this study proved that while caring for their children mothers continued to had an experience full of meanings or spirituality of life. The effects of spirituality in mothers’ lives was determined by changing the priorities, better managing daily routine, and following new options to face challenges which corresponds with some studies (Gray, 2006; Myers, Mackintosh & Goin-Kochel, 2009).

Having relationship with God considered being part of the spirituality of mothers with ASD children in this study. Also, it was used as an important source for solving their problems. Although some religious beliefs in spirituality and believe that, it’s excessive involvement causes negative aspects (Wortmann & Park, 2010), religion was recognized as an integral part of the spirituality of mothers of this study that defined as God’s deity. They felt closer to God after breeding sick child that accompany with praying and thanking God. As far as it was expected religious beliefs and practices formed spirituality and helped to reduce negative consequences of this phenomenon in mothers who participated in this study. In other words mothers searched ways to rescue their autistic children based on religious beliefs and using religious practices such as praise, pilgrimage, gratefulness and trust in God was the same as spirituality for them. Also, most mothers in this study believed in a connection between religious beliefs and spiritual values. They could help their children and also themselves based on religious and spiritual values. They believed in their ability to change and took steps toward better future for their children and also themselves and learned from pain and tribulation, how to care for their children in any possible way with tireless effort.

Even though some mothers have shown birth of an autistic child is considered as punishment from God (Tan et al., 2011), for our mothers it was not only a duty and gift that presented from God but also it was a good source of goodness and blessing in their lives. Most of participants in this study believed that the disability was coming from God and so they left all the problems to the love and the infinite wisdom of God. In contrary with different studies our mothers replaced submission and passiveness with dependence and trust in God (Ekas, Whitman, & Shivers, 2009; Tarakeshwar & Pargament, 2011).

Also, Mothers in this study believed that it was a very valuable job to do something worthwhile such as acceptance of the largest amount of responsibilities of caring for children with ASD. In this way, they believed that the only solution is to help their child (King et al., 2006) findings, they insisted on helping their children with doing as much effort as possible with the aim of the rescuing the child. There is a sense in mothers that they should deliver the child to the destination with any possible way. Indeed, helping her child was becoming everything in mother’s life.

Also, burden of problems and difficulties of caring for a child could not destroy the mother’s hope for having a better tomorrow. It was realized when they talked about their children’s improvements. Most participants were redounded with the sense of complacency and success. Despite of their awareness of the fact that this disorder was incurable of this they always kept a window of hope open for themselves (Koydemira & Tosuna, 2009; Hastings & Taunt, 2002).

Mothers in this study, who did not have any professional assistance, did a large part of managing problems and achieving goals in dealing for their children with following their inner- feelings (Emmons, 2000). Mothers allowed the problem solutions to follow their involvements.

Suffering related to the reality of children’s disorder and caring for them, has broken down the world of mothers. Although facing this reality took the meaning away from daily lives and gave them the feeling of loneness and being incapable, they could rely on their religious beliefs to find new meaning in their lives. After they found stability and they thought of new ideas in helping their children and they became more empowered through this purpose. Indeed, they reached a higher level of spirituality by caring for their children.

### 4.1 Strengths and Limitations

The phenomenological approach in this study was suitable to attain a deeper understanding of the mothers with an autistic child. Since mothers are more responsible for caring and their children in Iran’s culture, their experiences are more valuable. Then, the phenomenon under study becomes more important. The sample was obtained from a city in the Eastern part of Iran, so the findings may not be transfer to other parts of Iran with a different socio-cultural background.

## 5. Conclusion

This study carried out realizing the spiritual journey in mothers’ experiences who have caring for children with ASD. It could transform mothers’ meaning of lives and led them to look forward different ways to help their children and themselves. They considered the process of taking care of their children looked like something worthwhile and acceptance of this big responsibility prepared them to face challenges. Mothers who faced with this troublesome event used the spirituality in coping with problems, daily lives, caring their ASD children, and replace effort with surrendering. Indeed, Spirituality guided mothers of this study in the way of growing and refining their lives. It is important for nurses and other health care providers to know the meaning of mothers with an autistic child attributed to their caring experience. The findings of this study provide knowledge for nurses in order to help mothers deal with caring for children who suffer from autism spectrum disorders. Also, it can be share as positive outcomes of spiritual experiences of mother’s lives with others.
